# An Epidemiological Trend of Urogenital Schistosomiasis in Ethiopia

**DOI:** 10.3389/fpubh.2018.00060

**Published:** 2018-03-05

**Authors:** Bayissa Chala, Workineh Torben

**Affiliations:** ^1^Department of Applied Biology, School of Applied Natural Science, Adama Science and Technology University, Adama, Ethiopia; ^2^Department of Parasitology and Tropical Medicine, College of Medicine, Seoul National University, Seoul, South Korea; ^3^Division of Pathology, Tulane National Primate Research Center, Covington, LA, United States

**Keywords:** Ethiopia, prevalence, *Schistosoma haematobium*, urogenital schistosomiasis, snails

## Abstract

Schistosomiasis is a major public health problem in Ethiopia. Currently, the prevalence of the disease can possibly be heightened due to man-made ecological transformations particularly related to the recent development programs involving irrigation and construction of dams. The expansion of some of the water resource development projects has been cited enhancing the upsurge of urogenital schistosomiasis mainly in the lower altitude areas of the country. In connection to the extensive initiation of development projects in low altitude areas of the country, simultaneous and adequate attentions have never been given to address a pre-assessment of health impacts of the development programs prior to launching the projects. Helpful appraisals focusing on evaluation of epidemiology of urogenital schistosomiasis in Ethiopia have not been explored. Therefore, the current review attempts to trace an overall picture of the epidemiological status of urogenital schistosomiasis in the country; the past and existing trends of urogenital schistosomiasis surveys and control programs of the country are reviewed. Essential recommendations are highlighted for possible inputs in future control design strategies of national control program of schistosomiasis.

## Introduction

Human schistosomiasis is one of the most widespread parasitic infections in the world and found in 76 countries (1, 2). It is a chronic disease caused by blood flukes belonging to the genus *Schistosoma*. There are five schistosome species causing the disease: namely *Schistosoma haematobium (S. haematobium), S. mansoni, S. japonicum, S. mekongi*, and *S. intercalatum* (3). However, some studies report six *Schistosoma* species, including *Schistosoma guineensis*, which is related to *S. intercalatum* that is found in rainforest areas of central Africa (4). A number of animal schistosome species such as *S. margrebowiei* or *S. bovis* may also occasionally infect humans (5). Of all schistosomes, *S. haematobium, S. mansoni*, and *S. japonicum* have been reported to be the most prevalent trematodes worldwide. Geographically, *S. haematobium* is prevalent in Africa and Middle East; *S. mansoni* in Africa, South America, and Caribbean; and *S. japonicum* in China, Indonesia, and Philippines.

According to a recent WHO report, schistosomiasis is estimated to affect 249 million people worldwide, of which at least 224 million affected people live in sub-Saharan Africa (6). It ranks second only to malaria as the most common parasitic disease, killing an estimated 280,000 people each year in the African region alone (7). Reports of WHO in the past 3 years showed that an estimated 779 million people are at risk, with 240 million infected cases and more than 200,000 deaths occurring each year worldwide (8).

It has been estimated that about 54 million are infected and 393 million individuals are at risk of infection due to *S. mansoni* alone. Similarly, *S. haematobium* accounts for about 112 and 436 million individuals’ infection and risk of infection, respectively (9). Formerly known as urinary schistosomiasis, *S. haematobium* infection was recently renamed “urogenital schistosomiasis” in recognition that the disease affects both the urinary and genital tracts in up to 75% of infected individuals (10). Surprisingly, *S*. *haematobium* is responsible for nearly half of morbidity and about 150,000 deaths per year in sub-Saharan Africa where the disease is prevalent causing significant morbidity and mortality even as compared to *S. mansoni*.

It is an established fact that human schistosomiasis including urogenital schistosomiasis is caused by active skin penetration of cercaria which migrates via the bloodstream and eventually reside in the vesical vessels of the urogenital bladder. The adult female worms lay eggs that are deposited in the wall of the urogenital bladder (11, 12). The eggs release highly inflammatory antigens (13), triggering granuloma formation and a range of urothelial abnormalities and related signs such as hematuria, dysuria, and lesions of the bladder, kidney failure, and bladder cancer (14–16). Recently, a prevalence of 71.7% *S. haematobium* with 44.8% and 10.3% granulomas and calcifications of the bladder wall, respectively, was reported in Angola. The study also revealed about 3.4% vesical tumor classified as squamous cell carcinoma (17). *Schistosoma haematobium* has been classified as a class 1 carcinogen by the WHO/IARC (18) and an enormous body of evidence exists to support that assertion.

Ethiopia being one of the countries with high burden of schistosomiasis in general, urogenital schistosomiasis in particular has been known to be prevalent in northeastern part of the country following the Awash River Valley. There have also been reports in eastern part of the country around Wabi Shebele River and as well as in the western part of the country. Previous pilot prevalence studies have been documented in several parts of the country mainly focusing on school-aged children. However, despite the fact that many development projects are progressing in several low altitude areas of the country and creating suitable conditions for urogenital schistosomiasis, there has not been a study that critically reviewed the magnitude, risk factors, and trend of the past and present status of urogenital schistosomiasis in the country. Therefore, the current review attempts to trace past and present epidemiological status of urogenital schistosomiasis in the country.

## Risk Factors in the Transmission of Human Schistosomiasis

In the current issues of global warming and climatic change, the epidemiologies of temperature-dependent infectious diseases including schistosomiasis are rapidly changing. Human induced ecological transformations like dam construction and irrigation scheme developments are becoming the major risk factors for the resurgence of parasitic diseases. Many agro-industrial projects around the world have long been implicated for their strong association with parasitic disease outbreaks (19, 20).

Since 1950s and 1960s, several epidemiological investigations of both intestinal and urogenital schistosomiasis have been conducted in different parts of Ethiopia including new transmission foci. Some of the pilot studies suggested that the major factors associated with the prevalence of the disease in the country were extensive population movement and water resource development (21).

Population movement, rural to urban migration, forced displacement; migration of workers and the rise of ecotourism have all contributed to the increase in schistosomiasis (22). Seasonal migration of workers from endemic areas to irrigation projects or major hydroelectric power plants might potentially lead to introduction of the parasite into new environment. For several socio-economic, environmental, and geo-political related reasons of the country, there have been several mobile populations within and from the neighboring countries to Ethiopia. According to UNHCR (23) report, several migrants from neighboring countries including Eritrea, Somalia, South Sudan, and Sudan were received by Ethiopia in 2014 and 2015. Unless the refugees are managed with special consideration, the likelihood of outbreak of urogenital schistosomiasis can be still high. Furthermore, almost all refugee camps in the country are located in peripheral and lower altitude parts of the country where the probability of urogenital schistosomiasis transmission might dominate over intestinal schistosomiasis.

Transmission of urogenital schistosomiasis is dependent on availability of specific snail hosts and human activities with water contacts (24). Therefore, the risks and reemergence of urogenital schistosomiasis is attributed to the range of snail habitats stimulated by water development schemes like dam construction (25). It has also been suggested that the highly focal distribution of *S. haematobium* transmission is largely due to the non-susceptibility of bulinine snails to the endemic strain of the parasite in the country and low water temperatures in the highlands. Moreover, lack of information on intermediate snail host and parasite relationships and the ecology of proven and suspected snail hosts do not permit predictions on the spread of the endemic *S. haematobium* (26).

Altitude and temperature appear to be the major factors that affect the distribution of both *Schistosoma* species (*S. haematobium and S. mansoni*) in Ethiopia. It has been suggested that Dulshatalo, one of the urogenital schistosomiasis endemic areas in Ethio-Sudan border, exhibited high prevalence of urogenital schistosomiasis mainly because of its suitable lower altitude of less than 800 m above sea level (27, 28). Endemic *S. haematobium* appears to be confined in its distribution to lowlands below 800 m altitude as opposed to the transmission altitude of *S. mansoni* which is between 1300 and 2000 m altitude (26). It is suggested that altitude influences the distribution and transmission of parasitic diseases indirectly, through its effect on temperature, and through temperature’s effect on snail.

Water contact frequency and behavior were also reported to be among the major risk factors for the transmission of the infection as individuals may acquire the disease during contact with water containing cercariae of the parasite (29). There have been reports indicating that the highest prevalence of infections is found in school-aged children, adolescents and young adults (30). One possible justification is due to their frequent water contact that would make them more vulnerable to schistosomiasis, and hence this age group would be associated more frequently with schistosomiasis problems (31, 32). Other studies also suggested that domestic activities such as washing, fetching, fishing, and crossing rivers with bare foot showed significant association with the occurrence of schistosomiasis (33, 34). Recreational activities like swimming and poor hygiene also make children vulnerable to schistosomiasis (35).

## Distribution of Urogenital Schistosomiasis in Ethiopia

**Figure 1:**
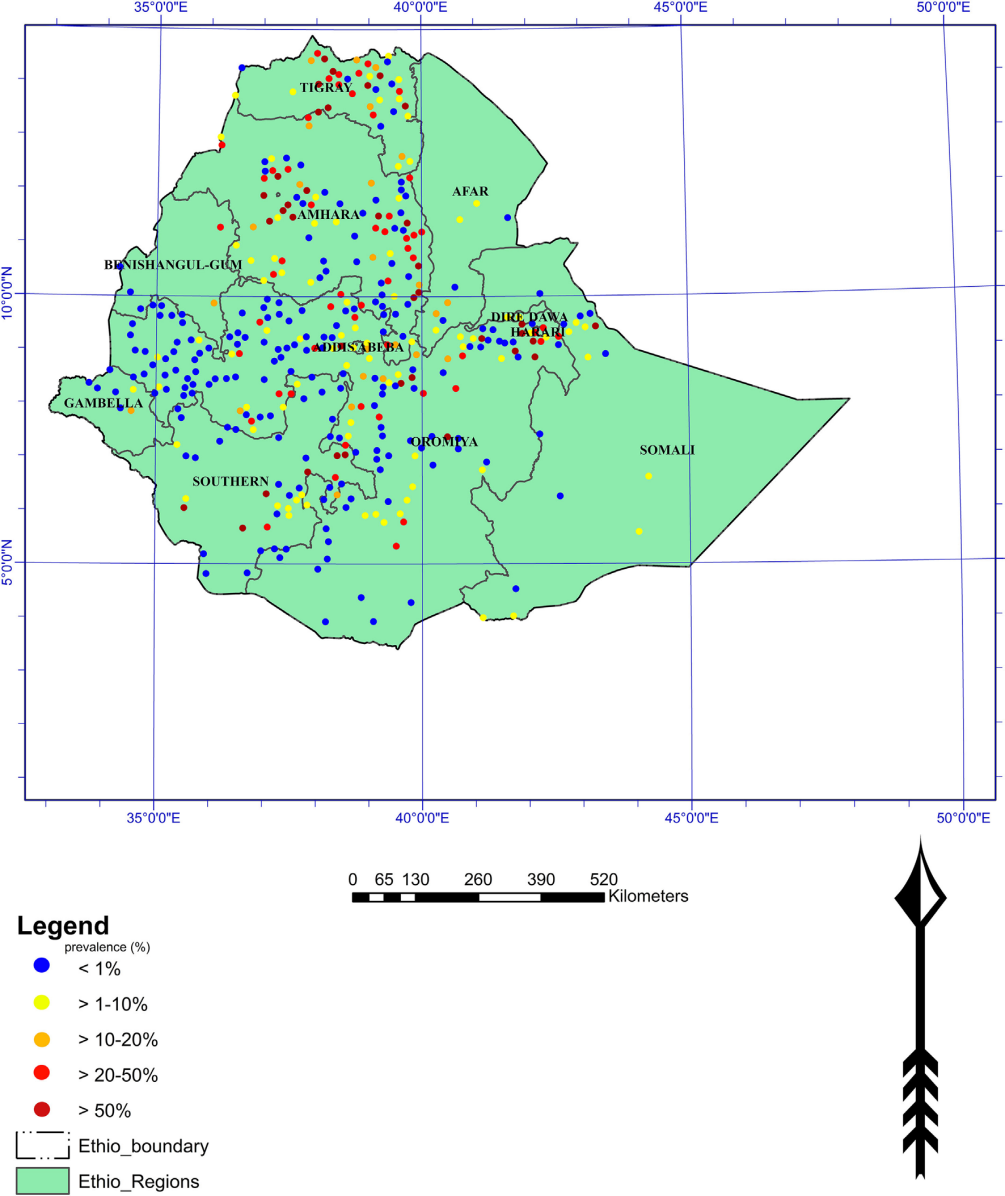
Distribution of schistosomiasis in Ethiopia (Adopted from “Global Atlas of Helminth Infections”).

Schistosomiasis is one of the most prevalent parasitic diseases of important public health problems in many developing countries including Ethiopia (Figure 1). It has been documented that Italian physicians in the northern part of the country first described the disease in the 1930s (36). Both *S. haematobium and S. mansoni* are endemic in Ethiopia, and an estimated 4 million people were infected and 30–35 million being at risk of infection (37). It has recently been reported that about 5.01 million people are thought to suffer from schistosomiasis and 37.5 million are at risk of getting the infection (38) (Figure 1).

Being the chief cause of urogenital schistosomiasis in Africa and the Arab world, *S. haematobium* is transmitted by *Bulinus* snails (3). The genus *Bulinus* comprises the following species: *B. tropicus, B. globosus, B. truncatus, B. forskalli*, and *B. africanus*. Urogenital schistosomiasis has been known to be endemic in several lower altitude areas of Ethiopia where it causes considerable public health problems, mainly among school-aged children. *B. abyssinicus* and *B. africanus* are the major snail intermediate hosts for *S. haematobium* in the country. It has been reported that lower altitudes ranging from 300 to 700 m were suitable for the establishment of these snails and maintenance of the parasite (28).

Urogenital schistosomiasis is the second dominant trematode causing human schistosomiasis in Ethiopia. Inadequate documented information can be one of the major reasons for the disease to continue undiagnosed in many parts of the country. Nevertheless, recently a helpful picture of the problem was presented in the Global atlas of helminths infection of the geographical distribution of *S. haematobium* in Ethiopia,^1^ and few studies also reported that the distribution of urogenital schistosomiasis in the country is highly focal and limited to lowland areas such as northeastern part of the country following the middle and lower Awash valley, in eastern part of the country around lower Wabe Shebele valleys and Kurmuk on the Ethio-Sudan border (39–43).

Based on a literature review of prevalence surveys for schistosomiasis in the country, *S. haematobium* cases were reported from 30 of 54 communities, 17 of the communities had infection rates between 14 and 75%. Similarly, out of 365 communities studied between 1961 and 1986 for *S*. *mansoni*, cases were reported from 225 (62%) and in 85 (23%) with prevalence range of 10 to 92% (29). Earlier studies reported that endemic *S. haematobium* is spatially confined to the distribution of the tropical African snail species *B. abyssinicus* in swamps in Awash and Wabe Shebele basins and apparently to only one locality where *B. africanus* occurs in a stream near Kurmuk on the Sudan border. High rates of *S. haematobium* infection, transmitted by the swampy-dwelling *B. abyssinicus* snail intermediate hosts were wide spread among pastoral Afar in the middle and lower parts of the valley (44). It has been reported that even though about 10 *Bulinus* species are expected to occur in Ethiopia, *B. abyssinicus* and *B. africanus* are the only bulinid species found naturally transmitting *S. haematobium* in the country (45).

The result of parasitological surveys in the Afar residents around the swamps and lakes in the middle part of the Awash Valley on the flood plains of the Awash River showed that *S. haematobium* infections are the most prevalent with infection rates being between 6–52 and 0–27% among seminomadic Afar and in agricultural groups, respectively (44). Similarly, a survey of the lower Wabi Shebelle Valley of southeastern Ethiopia showed that the infection due to *S. haematobium* is associated with the ecology of the areas, being highly common in downstream areas of Wabi Shebelle River (41). The development of irrigation schemes and harvesting projects in different swampy areas of the country has been shown to contribute to the spread of the disease (37).

For the past few decades, school-based epidemiological surveys have been the most frequent forms of parasitological studies throughout the country. Jemaneh et al. (46) had reported a high prevalence (70%) of urogenital schistosomiasis using filtration method in Amibara irrigation scheme (Hassoba). The use of questionnaire based approaches related to the clinical indicators of urogenital schistosomiasis such as blood in urine and pain when urinating was also suggested to be cost effective in urogenital schistosomiasis endemic area (47). However, there was no significant correlation between children’s and teachers’ questionnaire answers for the markers of urogenital schistosomiasis. Aemero (48) also has reported 46% prevalence of *S. haematobium* in Hassoba village using urine dipstick method. Similarly, Ayele et al. (49) reported 47.6% prevalence of urogenital schistosomiasis by urine filtration method among Hassoba village school children. More recent studies reported that school-aged children are the most affected groups, and growth retardation and poor school performance are adverse effects of the disease besides clinical manifestation and its complication (50).

A recent cross sectional school-based study on elementary school students in Amibera district, Ethiopia, showed that the overall prevalence of schistosomiasis in the area was 8.2% and *S. haematobium* and *S. mansoni* accounted for the average prevalence of 7.4 and 0.8%, respectively. Swimming habit and source of water for domestic consumptions were shown significant association with the occurrence of *S. haematobium* infection (51). Another prevalence survey of urogenital schistosomiasis in Amibara district, Afar region of Ethiopia was reported to be 24.54%. Moreover, the study showed that anemia is higher in concurrently infected children with urogenital schistosomiasis and malaria than non-infected and single infected (39). Another more recent epidemiological survey of *S. haematobium* among school-aged children living in the middle and lower Awash Valley, Afar regional state of Ethiopia was investigated. The overall prevalence was 20.8% which considerably varied across villages from 12.5 to 37.0% and 0 to 5.3% in the middle and lower Awash valleys, respectively (52). In general, previous studies in the Afar area documented a prevalence of *S*. *haematobium* ranging from 3.1 to 52.0% (39, 44, 46, 53).

Few studies on epidemiological aspects of urogenital schistosomiasis were conducted in low land areas including Somali regions of Ethiopia. Earlier reports showed that 30% of 50 male Ethiopian prisoners returning from Somalia were positive for *S. haematobium* eggs and 94 and 96% of the prisoners gave history for hematuria and treatment for schistosomiasis while in Somalia, respectively (54). More recently, it has been reported that the prevalence of *S. haematobium* in Afdera and Gode zones of Somali national regional state of Ethiopia was 16.0%, ranging from 11.8% in Kelafo to 64.2% in Musthail districts (55). This study also asserts that no infections of *S. mansoni* were found in these settings.

Similarly, another recent epidemiological study from Gambella Regional State, southwestern Ethiopia reported that the prevalence of urogenital schistosomiasis was 35.9% (109/304) with a mean egg intensity of 8.76 per 10 ml of urine. Based on the result, it has been recommended that treatment of all school-aged children and fishermen is required once every 2 years until the prevalence of infection falls below the level of public health importance (56). Likewise, a prospective study in Dulshatalo village, western Ethiopia near Ethio-Sudanese border, showed that the prevalence of *S. haematobium* among the study participants was 57.8% (197/341). In the same study participants, hematuria, one of the common clinical presentation of urogenital schistosomiasis was detected in 234 (68.6%) of the study participants (27).

## Control Measures and Challenges

Schistosomiasis has successfully been eliminated in Japan and Tunisia. Morocco and some Caribbean Island countries have made significant progress on controlling the disease while Brazil, China, and Egypt are taking steps toward elimination of the disease (57). In Asia the disease has largely been controlled by economic development and the filling and drainage of snail habitats, especially in Japan. On the other hand, the disease still exists in the Yangtze River basin, in China. Schistosomiasis is more rampant in poor rural communities, especially places where fishing and agricultural activities are dominant.

In some countries, schistosomiasis transmission may have been interrupted through active control programs and/or changing the socio-economic conditions. Schistosomes constitute serious public health problems in Ethiopia, with estimated nationwide prevalence of 16.5% (58). In countries like Ethiopia where schistosomiasis is endemic, control is aimed at reducing morbidity and arresting symptoms of the disease. In the 2013–2014 Ethiopian national mapping of schistosomes and STHs, variation in water, sanitation, and hygiene (WASH) assessed alongside the parasite infections for 80,475 children in 1,645 schools selected for the survey may reduce transmission of these parasites. The parasitological results showed prevalence of 3.5%, 0.2%, 13.3%, 7.8%, and 7.4%, for *S*. *mansoni, S*. *haematobium, A*scaris *lumbricoides, Trichuris trichiura*, and hookworm, respectively (59). Preventive chemotherapy (PC) can reduce morbidity caused by schistosomes (60). According to the assessment of population requiring PC report in 2010, despite Ethiopia’s endemicity for schistosomiasis, control of the disease is unfortunately still a long way to go. On top of that there is no recent mapping of the disease despite its crucial importance to guide health officials to distribute helpful control measures. Only limited NTDs including schistosome monitoring and control had taken place as shown in report on a national survey that took place in 1988–1989 in Ethiopia (61). Thus, mapping the distribution of schistosomiasis has been suggested as an important first step in establishing a national schistosomiasis control program in Ethiopia. It has been suggested that WASH in schools might improve attendance and educational attainment via preventing the transmission of schistosomes around and STHs within schools.

### Water Resource Developments and Their Implication for Schistosomiasis

Construction of dams and irrigation schemes are appropriate means in increasing food and energy demands of the growing world population. However, the associated infrastructure development projects have been implicated for stable transmission intensification or introduction of different vector-borne diseases including schistosomiasis into previously non-endemic areas (19, 20). Schistosomiasis is considered to be a sensitive indicator disease for monitoring ecological transformations, because it is widely distributed and its infection rates can change promptly (62). Likewise, it has been reported that environmental changes linked to water resource development and migrations can facilitate the spread of schistosomiasis to non-endemic areas (3).

Many surface irrigation systems in Africa create favorable snail-breeding conditions and subsequently facilitate the transmission of schistosomiasis. Agro-ecological regions in Morocco, Mali, Sudan, Cameroon, Egypt, Burkina Faso, Kenya, and Zimbabwe are among the few examples (63). For instance, there was an introduction of *S. mansoni* to Mauritania and Senegal after construction of huge Diama dam on the Senegal River (3) and Koka dam in Ethiopia (26). Similarly, the Nile dams in Sudan and Aswan dam in Egypt have been reported to worsen the existing transmission of urogenital schistosomiasis in the countries. It was recognized that the construction of Aswan dam in Egypt increased infection with urogenital schistosomiasis from less than 11% of the population in 1934 to 75% in 1937 (20).

Interestingly, previous studies demonstrated that there is a well-established relationship between development of irrigation and schistosomiasis. The studies mostly present evidences to elucidate the principle that water resource development projects have an “inevitable” adverse effect on health and the environment. Some studies have also recounted that development of irrigation schemes led to significant environmental modification, favoring the spread of vector-borne diseases, including schistosomiasis (38).

Similarly, it has long been reported that the two Koka dams of Awash Valley in Ethiopia have been associated with the transmission of *S. mansoni* and *S. haematobium* mainly through their downstream hydrology and irrigation but not the reservoirs (26). In addition, schistosomiasis has been rapidly spreading in connection with both water resource development and intensive population movements of the country (64). Currently, there are numerous small-scale and large-scale irrigations in different parts of the country, the majority being in lowlands or semi-lowland areas along river basins. Large scale irrigations are mainly state-owned projects. The mechanism of irrigation system can be either surface or sprinkler irrigation systems. The vast majority of the irrigation schemes employ the surface irrigation system. In line with this condition, some previous studies in Zimbabwe reported that more schistosomiasis risk factors were identified in surface irrigation schemes than sprinkler irrigation (65).

Both intestinal and urogenital schistosomiases have been widely reported in several regions of Ethiopia and the prevalence is significantly high. Despite high prevalence of the disease, there seems no specific integrated control policy launched to cutback the disease burden, especially in endemic areas of the country. It can be suggested that being one of neglected tropical diseases, disease burden due to schistosomiasis might be overlooked and more priorities have been given for high mortality disease like malaria, tuberculosis, and HIV/AIDS from the national control point of view. In comparison, schistosomiasis has low mortality but high morbidity rates that incapacitate the victims’ disability adjusted life years (DALYS) thereby increasing dependence and poverty complicating the livelihood of millions in most of the developing nations including Ethiopia.

### Chemotherapy As Control Option

The major challenges in schistosomiasis intervention remain the scale-up of treatment and the need to advocate for increased resources for implementation of treatment programs. PC was introduced by the World Health Organization aimed at using available anthelminthic drugs either alone or in combination as a public health tool for preventing morbidity due to more than one form of helminthiasis at once. For schistosomiasis, PC is only required in 52 endemic countries with moderate to high transmission, and the frequency of treatment is determined by the prevalence of infection in school-age children (6). Some WHO reports are showing promising results of PC against NTDs including schistosomiasis. On the other hand, eliminating diseases requires not only the right tools including drugs, but also the system to deliver them in a timely and efficient manner. There is also a need to model how these new tools would promote global elimination efforts for each of the major NTDs. For instance, recent reports from WHO World Health Report Research for Universal Health Coverage and related studies showed that all countries must become not only research users but also research producers (66, 67).

Deworming using praziquantel (PZQ) has been the most successful intervention method in the schistosomiasis endemic areas but lack of coordination and coverage of all endemic areas is the major weakness of the program. PZQ is administered on the basis of the WHO recommendations in a community level targeting schoolchildren experiencing high prevalence of schsitosomiasis. Such programs of mass treatment with PZQ are often performed annually (68). Intermittent mass treatment of school children with single dose of PZQ has been the most and successful common practice of intervention modality. If PC is coupled with provision of safe water, adequate latrines, and health education to decrease the risk of exposure and contaminative behavior, potentially a successful control and elimination of the disease was indicated (22).

Previous studies reported 85–100% reduction of local prevalence of intestinal schistosomiasis by mass chemotherapy and snail control in Blue Nile Valley of western (69). The efficacy of PZQ in Dulshatalo, western part of Ethiopia where urogenital schistosomiasis is endemic, showed a significant parasitological egg reduction rate and a decrease in hematuria and proteinuria 7 weeks post treatment. This indicated that implementation of mass chemotherapy can reduce the burden of the disease (27), which can subsequently minimize the disease transmission as well.

Beginning from year 2015, the Ethiopian Federal Ministry of Health with schistosomiasis control initiative (SCI) declared to launch national school-based deworming program to treat at risk school-aged children for intestinal worms and schistosomiasis across the country. Therefore, sustainable control of the disease requires integrated approach including repeated mass chemotherapy using PZQ, public health education focusing on behavior changes toward risk factors, improving sanitation, provision of clean water supply, and improving the life style of the community.

### Snail Vector Control Measures

The discovery of molluscicidal properties of Endod (*Phytolacca dodecandra*) by Ref. (41) has been a major breakthrough in combating intermediate snail hosts in schistosomiasis control in Ethiopia. Endod (*P. dodecandra*) has been evaluated in laboratory and field condition to be the most potent plant with molluscicidal activity against snail intermediate hosts of schistosome (70).

The most effective method to significantly reduce snail population density is the use of molluscicide like niclosamide, pesticides specifically made to terminate aquatic snails. Application of niclosamide is recommended to be seasonal based on transmission patterns targeting active transmission areas (71). Alternative approaches that may include better strategies for molluscicide application, environmental and biological interventions are needed to reduce the application of niclosamide and to provide more long-lasting effects.

The use of plant molluscicide has been considered as an alternative way to control snail populations. Endod-based snail control should also be the part of these chemical control approaches. However, snail control by itself may not be the magic bullet in the control of schistosomiasis. In Ethiopia, berries of Endod (*P. dodecandra*) were formerly used extensively as soap for washing laundry. Experiments have been underway to determine how the introduction of Endod in snail-infested locations can contribute to schistosomiasis control (72). Endod-based *Biomphalaria pfeifferi* snail control in northeastern part of Ethiopia suggested that Endod-based molluscicide control approach in conjunction with chemotherapy can be effective in the control of schistosomiasis (73). Similar study reported a progressive decline in the *B. pfeifferi* snail population and infection as a result of implementation of Endod-based snail control (74). Presumably, the Endod-based snail control approach can also be extended in the same way to *Bulinus* snails genera, transmitting *S. haematobium*. On the other hand, perhaps due to the relatively high cost of molluscicides, public health intervention measures have not been encouraged that much to use these local molluscicidal plant for routine purposes. Furthermore, preference for commercial soap and lack of land for cultivation are major obstacles for increasing the availability and use of Endod. In addition, an iniquitous association of Endod use and low social status makes many people uninterested in production and sale, notably in the western and south-central parts of the country (75).

### Health Impact Assessment of Water Development Projects

Human-made ecologic transformations have been seen at an unprecedented rate over the past six decades. The prominent changes are related to water resource development projects and irrigations involving small and large dams worldwide. The complex health impact of the constructions associated with dams and the downstream sites was well reported in previous studies (76). The establishment and operation of water-related projects have a proven history of facilitating a change in the frequency and transmission dynamics of vector-borne diseases including schistosomiasis.

Recently, agricultural development and water harvesting programs of irrigation have been intensified in Ethiopia. Most importantly, sugarcane plantations and cotton farming are the most commonly agro-industrial activities associated with the transmission of schistosomiasis. Other than agro-industrial activities, extensive and large scale farming of sesame, vegetables, and and fruits in different semi-arid localities of the country utilizes irrigated water systems with potential link with schistosomiasis. However, preliminary investigations indicated that preparations of the irrigation system in various part of Ethiopia appeared to miss its health impacts (77). Addressing the adverse impact of agricultural water projects on both health and water sectors tend to focus on economic benefits paying inadequate attention to assessing public health and environmental impact. Mainly, water projects have been planned and managed separately instead of linking with other related progresses at the local, district, and national levels. Despite the fact that several irrigation based agro-industrial projects and hydroelectric power generating dams including the Grand Ethiopian Renaissance and Gilgel Gibe hydroelectric power constructions are in progress, no specific pre-assessment control strategy that targets schistosomiasis has been considered in the country. However, the development projects will inevitably result in the spread of the disease unless careful preparation is made for appropriate interventions in areas where transmission has already been established. Suitable health impact assessment and prevention strategies should be contemplated prior to initiating new water resource and hydroelectric power plants.

## Future Control Directions

Basic pilot studies should be promoted in various endemic areas of the country to estimate the level of prevalence and associated risk factors in the areas. Such epidemiological surveys may contribute a lot in mapping of active transmission sites of the disease. It has been suggested by scholars that mapping of the risk areas is an important first step in establishing a national schistosomiasis control program.

Currently, in most schistosomiasis endemic sub-Saharan African countries, there are partnership movements and interests to assist governments to establish countrywide elimination programs. Among the major partner institutions targeting the elimination and control of schistosomiasis as well as soil-transmitted helminths and other NTDs in Ethiopia are Carter Center, SCI, and UNICEF. These partner institutes provide resources for implementation of treatment programs, provision of potable water, adequate sanitation, hygiene education, and snail control. Furthermore, experience of countries like Egypt where intervention programs led to a dramatic decrease of schistosomiasis should be considered. Endod-based molluscicide control approaches should also be given proper attention and as an alternative control measure in conjunction with PC.

## Concluding Remarks and Recommendations

Ethiopia is one of the countries with high burden of schistosomiasis mostly due to *S. mansoni* and *S. haematobium* nevertheless there is no strong and coordinated control program in the country. The past and present epidemiological studies showed that urogenital schistosomiasis is highly prevalent mostly in the river basins of lower altitude areas of the country. The infection rate though differs for different study areas, as high as 75% prevalence was reported. Mainly, several epidemiological studies were exclusively targeted and conducted on school-aged children and few surveys included adults of the communities. This can be inadequate in determining the level of prevalence of the infection in the target communities. On top of that it cannot be easy to imagine age-related pattern and trend of the disease in a specified location. Therefore, in collaboration with partnership agents, national wide prevalence survey including all age groups should be initiated to better estimate the magnitude of the disease in the country.

## Author Contributions

Both authors contributed equally to the manuscript.

## Conflict of Interest Statement

The authors declare that the research was conducted in the absence of any commercial or financial relationships that could be construed as a potential conflict of interest.

## Footnotes

^1^http://www.thiswormyworld.org/maps/et.
